# Nerve pathology is prevented by linker proteins in mouse models for *LAMA2*-related muscular dystrophy

**DOI:** 10.1093/pnasnexus/pgad083

**Published:** 2023-03-15

**Authors:** Judith R Reinhard, Emanuela Porrello, Shuo Lin, Pawel Pelczar, Stefano C Previtali, Markus A Rüegg

**Affiliations:** Biozentrum, University of Basel, 4056 Basel, Switzerland; Neuromuscular Repair Unit, InSpe and Division of Neuroscience, IRCCS Ospedale San Raffaele, 20132 Milano, Italy; Biozentrum, University of Basel, 4056 Basel, Switzerland; Center for Transgenic Models, University of Basel, 4056 Basel, Switzerland; Neuromuscular Repair Unit, InSpe and Division of Neuroscience, IRCCS Ospedale San Raffaele, 20132 Milano, Italy; Biozentrum, University of Basel, 4056 Basel, Switzerland

**Keywords:** laminin, merosin, radial sorting, myelination, MDC1A

## Abstract

*LAMA2*-related muscular dystrophy (LAMA2 MD or MDC1A) is a devastating congenital muscular dystrophy that is caused by mutations in the *LAMA2* gene encoding laminin-α2, the long chain of several heterotrimeric laminins. Laminins are essential components of the extracellular matrix that interface with underlying cells. The pathology of LAMA2 MD patients is dominated by an early-onset, severe muscular dystrophy that ultimately leads to death by respiratory insufficiency. However, pathology in nonmuscle tissues has been described. Prior work in the *dy^W^*/*dy^W^* mouse model for LAMA2 MD has shown that two linker proteins, mini-agrin and αLNNd, when expressed in skeletal muscle fibers, greatly increase survival from a few months up to more than 2 years. However, the restoration of skeletal muscle function accentuates the pathology in nonmuscle tissue in *dy^W^*/*dy^W^* mice, first and foremost in the peripheral nerve resulting in paralysis of the hind limbs. We now show that the expression of the two linker proteins in all tissues ameliorates the muscular dystrophy and prevents the appearance of the hind limb paralysis. Importantly, the same ameliorating effect of the linker proteins was seen in *dy^3K^*/*dy^3K^* mice, which represent the most severe mouse model of LAMA2 MD. In summary, these data show that the two linker proteins can compensate the loss of laminin-α2 in muscle and peripheral nerve, which are the two organs most affected in LAMA2 MD. These results are of key importance for designing appropriate expression constructs for mini-agrin and αLNNd to develop a gene therapy for LAMA2 MD patients.

**Significance Statement**

*LAMA2*-related muscular dystrophy (LAMA2 MD) is the most frequent congenital muscular dystrophy that leads to early death with no available treatment. The disease is caused by loss-of-function mutations in the large *LAMA2* gene encoding the laminin-α2 chain of the extracellular matrix (ECM) heterotrimeric protein laminin-211. Prior work showed that two small linker proteins, designed from domains of other ECM constituents, functionally compensate for the loss of laminin-211 in skeletal muscle of LAMA2 MD mice. We now show that the linkers can restore function in nonmuscle tissues. Most strikingly, hind limb paralysis is completely prevented in LAMA2 MD mice. Our results are a crucial step for the selection of the proper target tissue and promoter for future gene therapies for LAMA2 MD patients.

## Introduction

Skeletal muscle is required to maintain posture, for locomotion, respiration, and overall metabolic homeostasis. Motor neurons originating in the spinal cord control the contraction of muscle fibers by innervating them at the neuromuscular junction where they release acetylcholine to trigger an action potential in the muscle fiber to cause its contraction. At the same time, limb position and movement, tension, and force of muscle fibers are sensed by the proprioceptive system, which feeds this information back to the spinal cord. Thus, skeletal muscle function requires a complex network that sends impulses from the spinal cord to muscle and back again via peripheral nerves (1). To allow fast conductance of the action potentials, motor and proprioceptive axons are myelinated by Schwann cells.

Congenital muscular dystrophies (CMDs) are characterized by early onset (diagnosed around birth), high severity, and reduced life span. *LAMA2*-related muscular dystrophy (LAMA2 MD or MDC1A) is caused by mutations in *LAMA2*, the gene coding for the laminin-α2 chain of the heterotrimeric laminins, composed of α, β, and γ chains (2, 3). In total, 16 different laminins (i.e. heterotrimers with different chain compositions) have been described in mammals. The most abundant, laminin-α2–containing heterotrimer is laminin-211 (formerly called merosin), composed of the α2, the β1, and the γ1 chains. In mice, laminin-211 is mainly detected in the endomysial basement membrane (BM) of mature skeletal muscle fibers, in the heart, and in the endoneurial BM of the peripheral nerve (4). Consistent with such expression pattern, the disease in complete or partial laminin-α2–deficient patients is dominated by the muscular dystrophy. In contrast to the early-onset CMD patients (associated with complete laminin-α2 deficiency), partial deficiency causes a more variable, milder clinical presentation (5). Some of these patients display a limb–girdle muscular dystrophy-like phenotype (6–8) that is classified as “LGMD R23 laminin α2-related” (9). In some patients, case reports have also described heart involvement (10, 11) and peripheral neuropathy (12). Hence, there is evidence that LAMA2 MD is not a pure muscular dystrophy. This is accentuated in LAMA2 MD mouse models where the peripheral neuropathy manifests as a progressive hind limb paralysis (13).

Skeletal muscle from LAMA2 MD patients and mouse models “compensates” the loss of laminin-α2 by expression of the embryonic laminin-α4 chain (14, 15). Laminin-α4–containing laminin-411 does not bind to the bona fide cell surface receptors of laminin-211 and is not able to self-polymerize (15, 16) and thus is not able to functionally compensate. In an attempt to “graft” both functions of laminin-α2 onto laminin-411, two linker proteins have been designed that consist of domains of agrin [mini-agrin (mag)] or laminin-α1 and nidogen-1 (αLNNd). Both proteins bind to laminin-411; mag, in addition, binds to α-dystroglycan and αLNNd allows laminin-411 to polymerize (17, 18). Muscle-specific, transgenic expression of either mag or αLNNd in different LAMA2 MD mouse models [i.e. *dy^W^*/*dy^W^*, *dy^3K^*/*dy^3K^*, or *dy^2J^*/*dy^2J^* mice (19)] ameliorates the disease (17, 20, 21). Importantly, transgenic expression of both linker proteins in skeletal muscle fibers of *dy^W^*/*dy^W^* mice provides additive benefit, leading to a drastic prolongation of the median life span from less than 4 months to more than 18 months (15). However, the long survival accentuates the progressive peripheral neuropathy in *dy^W^*/*dy^W^* mice (15), as was also observed upon muscle-specific, transgenic expression of laminin-α2 (22).

In the current work, we tested whether the linker proteins could ameliorate the muscular dystrophy and prevent hind limb paralysis when expressed ubiquitously. To this end, we generated novel transgenic mice for the two linker proteins and evaluated their disease ameliorative effect in *dy^W^*/*dy^W^* and *dy^3K^*/*dy^3K^* mice. We report here that ubiquitous expression of the two linker proteins restores both muscle and peripheral nerve functions resulting in near-normal body weight, gait, and muscle force. These data thus provide unequivocal evidence that linker proteins can functionally replace laminin-211 in muscle and in the peripheral nerve.

## Results

### Sciatic nerve of LAMA2 MD mice contains laminin-α4 and linker proteins localize to the endoneurial BM when expressed ubiquitously

**Fig. 1.**
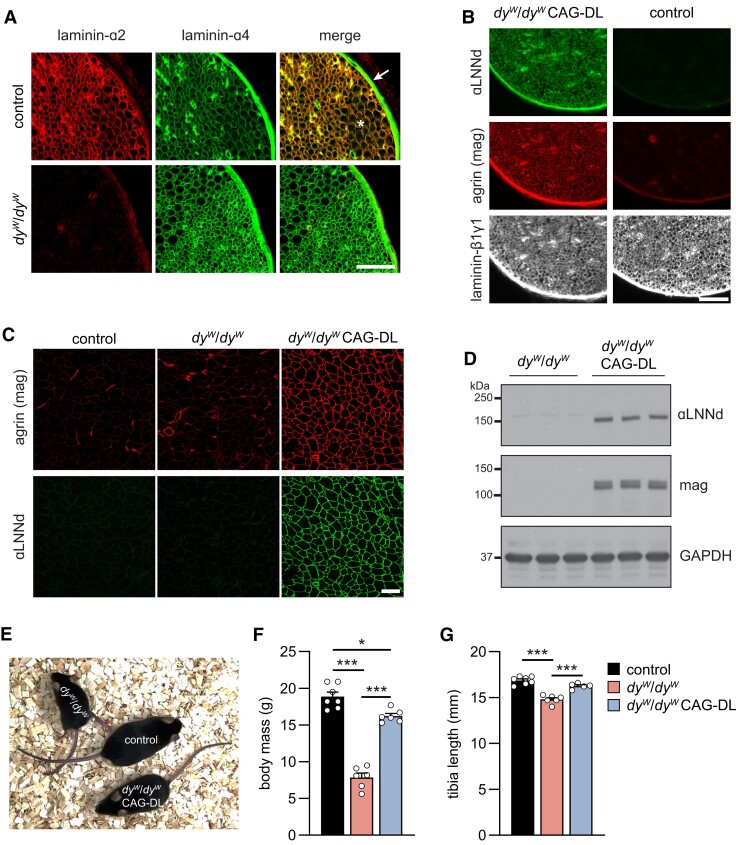
Expression of laminins and linkers in control and LAMA2 MD mice. A) Cross-sections of the sciatic nerve from control and *dy^W^*/*dy^W^* mice stained with antibodies to laminin-α2 and laminin-α4. Note the increase in laminin-α4 immunoreactivity in the endoneurium in *dy^W^*/*dy^W^* mice. Arrow indicates the perineurium and asterisk the endoneurium. B, C) Cross-sections of the sciatic nerve (B) and TA muscle (C) of 8-week-old mice of the indicated genotype. The linker proteins αLNNd and mag are present in the endoneurium and perineurium of the sciatic nerve (B) and in the muscle fiber basement membrane (C). D) Western blot analysis to detect αLNNd and mag in lysates from *triceps brachii* muscle of 8-week-old mice. Glyceraldehyde 3-phosphate dehydrogenase (GAPDH) was used as loading control. E) Overall phenotype of 14-week-old mice of the indicated genotype. F, G) Body mass (F) and tibia length (G) of 8-week-old mice of the indicated genotype. Data are mean ± SEM. *P < 0.05 and ***P < 0.001, by one-way ANOVA with Bonferroni post hoc test. Scale bars: 50 *µ*m (A, B) and 100 *µ*m (C). *N* = 5–7 female mice per group.

LAMA2 MD mouse models and LAMA2 MD patients express the compensatory laminin-411 in the muscle BM (14, 15). As the ameliorative function of mag and αLNNd in *dy^W^*/*dy^W^* mice requires their binding to laminin-411, we stained sciatic nerve cross-sections with antibodies to laminin-α2 and laminin-α4 (Fig. 1A). The endoneurial, but not the perineurial BM was strongly positive for laminin-α2 in control mice. In contrast, laminin-α4 staining was weak in the endoneurial but strong in the perineurial BM. In *dy^W^*/*dy^W^* mice, laminin-α4 staining was strong in both endoneurial and perineurial BM (Fig. 1A).

To explore the possibility that expression of the two linker proteins mag and αLNNd would also ameliorate nonmuscle phenotypes in *dy^W^*/*dy^W^* mice, we inserted the sequences coding for mouse mag or αLNNd into the *Rosa26* locus of C57BL/6J mice by CRISPR/Cas9-driven homology-directed repair (for a schematic presentation see Fig. S1A). In these knockin mice, upon Cre-mediated deletion of the stop cassette, the linker proteins are expressed by the hybrid construct consisting of the cytomegalovirus (CMV) enhancer fused to the chicken beta-actin (CAG) promoter. To test the functionality of the cloned coding sequences, we transfected COS7 cells with the targeting vectors. Only upon co-transfection with a Cre-expressing plasmid, we detected mag or αLNNd in cell extracts (top rows) and the culture medium (bottom rows) (Fig. S1B). This confirms the functionality of both constructs and shows that the loxP–Stop–loxP (LSL) cassette prevents any leakage. To select the appropriate Cre system, we crossed different mouse lines with tdTomato reporter mice [Rosa26-CAG-LSL-tdTomato (23)], among them the transgenic CAG-Cre-ER mice that express a tamoxifen-responsive Cre recombinase under the CAG promoter (24). Examination of skeletal muscles showed that CAG-Cre-ER caused strong Tdtomato expression (observed as red coloring of the entire muscle), irrespective of tamoxifen injection (Fig. S1C). These experiments show that some recombination of the LSL cassette occurred in CAG-Cre-ER–positive mice even in the absence of tamoxifen, as previously reported (24), eventually sufficient for linker protein expression. Indeed, triple transgenic mice, not injected with tamoxifen, that were positive for the CAG-Cre-ER transgene and carried one copy of the Rosa26-CAG-LSL-mag and one copy of Rosa26-CAG-LSL-αLNNd allele (called CAG-DL for CAG-driven Double Linker) expressed both linker proteins from birth onwards with some difference in the expression levels between individual mice in the first 2 weeks (Fig. S1D). The deletion of the LSL cassette in Cre-positive mice in brain and skeletal muscle was also confirmed on the genomic level (Fig. S1E). By further intercrossing, we generated *dy^W^*/*dy^W^* CAG-DL mice and compared them with *dy^W^*/*dy^W^* and control mice. None of the mice was injected with tamoxifen.

In *dy^W^*/*dy^W^* CAG-DL mice, the endoneurial and the perineurial BM was strongly positive for both linker proteins (Fig. 1B) as was the muscle BM (Fig. 1C), indicating that the linker proteins are expressed and incorporated into the BM. Western blot analysis of muscle lysates from 8-week-old mice confirmed expression of the linker proteins in *dy^W^*/*dy^W^* CAG-DL mice (Fig. 1D). The expression of the linker proteins induced a striking overall phenotypic improvement in adult *dy^W^*/*dy^W^* mice, to the extent that it became difficult to distinguish controls from *dy^W^*/*dy^W^* CAG-DL mice (Fig. 1E). Although the body mass of *dy^W^*/*dy^W^* CAG-DL mice was not different to *dy^W^*/*dy^W^* mice at the age of 3 weeks (Fig. S1F), they caught up with control mice and reached a body mass and size that was close to normal at 8 weeks of age (Fig. 1F and G).

### Linker proteins prevent hind limb paralysis by restoring axonal sorting and myelination

**Fig. 2.**
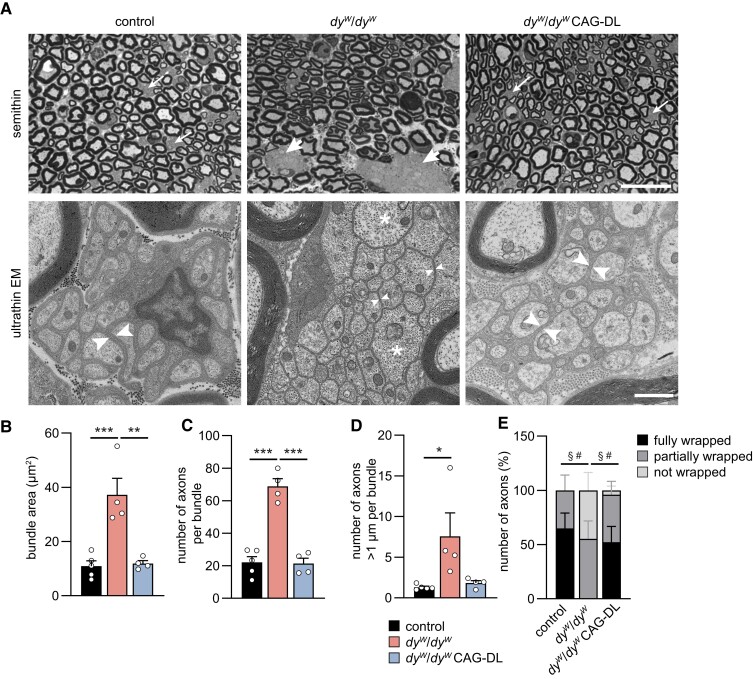
Ubiquitous expression of the linker proteins improves nerve pathology. A) Images of semithin and ultrathin cross-sections of the sciatic nerve from 8-week-old mice of the indicated genotype. Semithin: regions/bundles containing nonmyelinated axons are indicated with arrows. In *dy^W^*/*dy^W^* mice, the bundles are large (thick arrows), while bundles are small in control and *dy^W^*/*dy^W^* CAG-DL mice (thin arrows). Ultrathin EM: axons within the bundles in *dy^W^*/*dy^W^* mice are not ensheathed by Schwann cells (small arrowheads) and can be >1 µm in diameter (asterisks). In control mice, bundles represent Remak bundles of slow-conducting axons that are ensheathed by nonmyelinating Schwann cells (big arrowheads). In *dy^W^*/*dy^W^* CAG-DL mice, nonmyelinated axons are also surrounded by nonmyelinating Schwann cell processes (big arrowheads), reminiscent of Remak bundles in control mice. B–D) Quantitative assessment of the bundles in the different genotypes by measuring the area (B), the number of axons (C), and the number of axons >1 µm in diameter (D). E) Quantification of the extent of Schwann cell wrapping of axons in bundles in the different genotypes. Data are mean ± SEM. *P < 0.05, **P < 0.01, and ***P < 0.001, by one-way ANOVA with Bonferroni post hoc test (B–D). ^§^*P* < 0.05 in fully wrapped axons and ^#^*P* < 0.05 in not wrapped axons, by one-way ANOVA with Bonferroni post hoc test. Scale bars: 20 *µ*m (semithin) and 1 *µ*m (ultrathin). *N* = 4–5 mice per group.

We also noticed that in contrast to *dy^W^*/*dy^W^* mice and *dy^W^*/*dy^W^* mice that express the linker proteins only in skeletal muscle fibers (15), *dy^W^*/*dy^W^* CAG-DL mice did not display paralysis of the hind limbs (Movie S1). The peripheral neuropathy in LAMA2 MD mouse models is caused by severe defects in axonal sorting and myelination of motor and sensory axons (13, 25). Staining of semithin cross-sections of the sciatic nerve with toluidine blue revealed many bundles or islands of “naked” axons in *dy^W^*/*dy^W^* mice (thick arrows in Figs. 2A and S2A). These large regions of nonmyelinated axons were also a striking feature in the sciatic nerve of *dy^W^*/*dy^W^* mice in ultrathin, electron microscopic (EM) pictures (Fig. 2A). While these bundles were usually large in *dy^W^*/*dy^W^* mice, there were only few of them in *dy^W^*/*dy^W^* CAG-DL mice and they were smaller (Figs. 2A and S2A). Axons of *dy^W^*/*dy^W^* mice in the bundles were naked and not wrapped by Schwann cells (small arrowheads in *dy^W^*/*dy^W^* panel of Fig. 2A) and they contained many large-caliber axons (asterisks in *dy^W^*/*dy^W^* panel of Fig. 2A). In contrast, bundles of “naked” axons in *dy^W^*/*dy^W^* CAG-DL mice and controls were reminiscent of the Remak bundles (thin arrows in the semithin sections of Fig. 2A), which are small axons that are ensheathed by nonmyelin-forming Schwann cells (big arrowheads of the ultrathin EM panels of control and *dy^W^*/*dy^W^* CAG-DL mice of Fig. 2A). In *dy^W^*/*dy^W^* mice, quantification demonstrated a significant increase in the area of the bundles (Fig. 2B), in the number of axons within such bundles (Fig. 2C), in the number of large-caliber axons (Fig. 2D), and a significant decrease in the relative number of axons fully wrapped by Schwann cells (Fig. 2E) compared with controls. Importantly, all these parameters were restored to control values in *dy^W^*/*dy^W^* CAG-DL mice. We also observed an increase in the median axon diameter of myelinated axons in *dy^W^*/*dy^W^* mice compared with controls, and this diameter was again normalized in *dy^W^*/*dy^W^* CAG-DL mice (Fig. S2B). The *G*-ratio, an index of myelination, did not significantly change between *dy^W^*/*dy^W^* and *dy^W^*/*dy^W^* CAG-DL mice (Fig. S2C and D). Overall, these data demonstrate that expression of the two linker proteins in the peripheral nerve largely restores axonal sorting, a process that is highly perturbed in all mouse models of LAMA2 MD, irrespective of the mutation in *Lama2* (19).

### Linker proteins improve muscle function in *dy^W^*/*dy^W^*

**Fig. 3.**
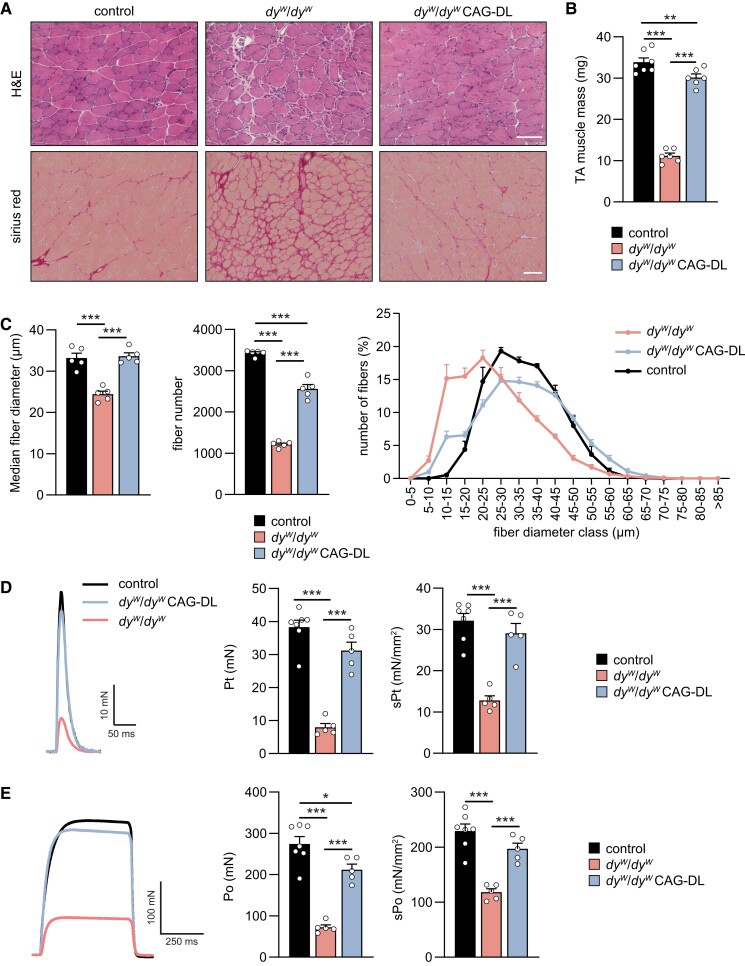
Linker proteins improve muscle histology and function in fore- and hind limbs of *dy^W^*/*dy^W^* mice. A) H&E and Sirius Red staining of 8-week-old TA muscle of the indicated genotypes. B) Quantification of the TA mass from 8-week-old mice of the indicated genotypes. C) Quantification of TA muscle fiber diameter and number from 8-week-old mice of the indicated genotypes. D, E) Twitch (D) or tetanic force (E) of EDL muscle from 8-week-old mice of the indicated genotypes. Shown are representative force traces (left), absolute (middle), and specific (right) force. Data are mean ± SEM. *P < 0.05, **P < 0.01, and ***P < 0.001, by one-way ANOVA with Bonferroni post hoc test. Scale bars: 100 *µ*m. *N* = 5–7 female mice per group.

To see whether the linker proteins improved muscle histology and function, we next examined fore- and hind limb muscles of 8-week-old mice. Quadriceps, gastrocnemius, and *triceps brachii* (TRC) muscles very significantly increased in mass in *dy^W^*/*dy^W^* expressing the linker proteins (Fig. S3A). Similar to the results obtained with skeletal muscle-specific expression of the linkers (15), *triceps brachii* muscle of *dy^W^*/*dy^W^* CAG-DL mice was strongly improved in median fiber diameter, fiber size distribution, and fibrosis but contained many fibers with centralized nuclei (Fig. S3B–D). The high number of fibers with centralized nuclei was also observed in *dy^W^*/*dy^W^* mice that express the two linker proteins in skeletal muscle (15) and may, at least in part, be due to the improvement of muscle regeneration by the linker proteins (26). Importantly, absolute and specific forelimb grip strength was increased such that *dy^W^*/*dy^W^* CAG-DL developed more than twice the force of *dy^W^*/*dy^W^* mice (Fig. S3E). Assessment of histology in *tibialis anterior* (TA) muscle, which is affected by the hind limb paralysis, showed strong overall improvement in hematoxylin and eosin (H&E) staining and in the extent of fibrosis, stained by Sirius Red, in *dy^W^*/*dy^W^* CAG-DL mice compared with *dy^W^*/*dy^W^* mice (Fig. 3A). The mass of the TA almost tripled in *dy^W^*/*dy^W^* CAG-DL mice compared with *dy^W^*/*dy^W^* mice (Fig. 3B). This increase was based on an increase in the median fiber size and in the total fiber number in *dy^W^*/*dy^W^* CAG-DL mice, reaching close-to-normal values (Fig. 3C). Ex vivo force measurement of the *extensor digitorum longus* (EDL) and *soleus* hind limb muscle showed a significant improvement by the linker proteins (Table S1). In EDL, the absolute and specific twitch force in *dy^W^*/*dy^W^* CAG-DL mice reached close-to-normal levels (Fig. 3D). Similarly, absolute and specific tetanic force was improved in *dy^W^*/*dy^W^* CAG-DL mice (Fig. 3E). This almost complete restoration of EDL muscle force in *dy^W^*/*dy^W^* CAG-DL mice is substantially better than in *dy^W^*/*dy^W^* mice that express the linker proteins only in skeletal muscle (15), suggesting that this reflects the ability of *dy^W^*/*dy^W^* CAG-DL mice to keep the hind limbs moving and prevent atrophy caused by immobility. Together, these data show that ubiquitous expression of the two linker proteins in *dy^W^*/*dy^W^* mice largely prevents the muscular dystrophy and the neuropathy-caused loss of muscle mass and force in EDL muscle.

### Profound motor improvement by linker proteins is sustained in different LAMA2 MD mouse models

**Fig. 4.**
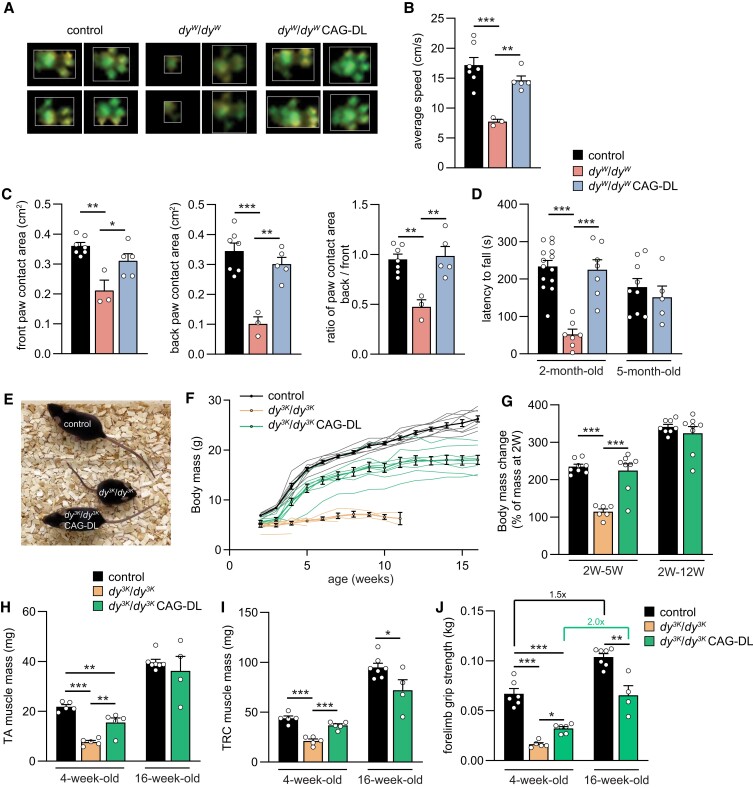
Motor performance of *dy^W^*/*dy^W^* and evaluation of the treatment effect in *dy^3K^*/*dy^3K^* mice. A) Representative paw prints of mice of the indicated genotypes using a gait analysis system (CatWalk). B, C) Quantitative assessment of locomotor speed (B) and paw contact area (C) using a gait analysis system (CatWalk). D) Rotarod-based assessment of motor function and coordination in 2- and 5-month-old mice of the indicated genotype. Note that *dy^W^*/*dy^W^* mice are not included at 5 months because they usually die before. E) Overall phenotype of 4-week-old mice of the indicated genotype. F) Body mass curves of female mice from 2 to 15 weeks of age of the indicated genotype. Curves for individual mice are shown including the mean ± SEM of each cohort (thick line). Body mass curves for individual *dy^3K^*/*dy^3K^* mice are discontinuous as all of the mice died at the age between 3 and 11 weeks. G) Relative increase in body mass from 2 weeks of age until 5 and 12 weeks, respectively. Note that weight gain in *dy^3K^*/*dy^3K^* CAG-DL mice up to 12 weeks is similar to controls. H, I) Muscle mass of TA and TRC of female mice at different ages. J) Forelimb grip strength of female mice of the indicated genotypes at 4 and 16 weeks of age. Data are mean ± SEM. *P < 0.05, **P < 0.01, and ***P < 0.001, by one-way ANOVA with Bonferroni post hoc test. Scale bars: 5 mm. *N* = 3–14 mice per group.

To assess the functional improvement in peripheral nerves of *dy^W^*/*dy^W^* CAG-DL mice, we performed quantitative assessment of locomotion and gait (Fig. 4A). Average speed of *dy^W^*/*dy^W^* mice was less than half of that of controls but significantly improved by the expression of the two linker proteins (Fig. 4B). To get an estimate on the relative use of front- and hind limbs, we analyzed the weight put onto each leg by measuring the contact area of the paws (Fig. 4C). In control and *dy^W^*/*dy^W^* CAG-DL mice, front and back paw contact areas were of similar size, whereas they were much smaller in *dy^W^*/*dy^W^* mice (due to the reduced body mass and size). To control for this body mass and size difference, we compared the ratio of the contact area of the back versus front paws. While the ratio was close to one in control and *dy^W^*/*dy^W^* CAG-DL mice, it was significantly reduced in *dy^W^*/*dy^W^* mice (Fig. 4C). Thus, hind limb paralysis prevents loading of the hind legs in *dy^W^*/*dy^W^* and thus reduces the relative contact area, while *dy^W^*/*dy^W^* CAG-DL and control mice use all limbs similarly. Finally, motor coordination and muscle function were assessed by the rotarod assay. Eight-week/two-month-old *dy^W^*/*dy^W^* mice showed a very short latency to fall, while control and *dy^W^*/*dy^W^* CAG-DL mice managed to stay on the rod for a similar time (Fig. 4D). Importantly, the performance of *dy^W^*/*dy^W^* CAG-DL mice remained similar to control mice at 5 months of age (Fig. 4D, right). Together, these data show that the ameliorating effect of the two linker proteins in the peripheral nerve allows for a close-to-normal locomotor function.

Next, we tested whether the expression of the two linker proteins would also improve the phenotype (including the peripheral neuropathy) in the most severe mouse model for LAMA2 MD by crossing the CAG-DL triple transgene combination into *dy^3K^*/*dy^3K^* mice (27). Like *dy^W^*/*dy^W^* mice, the dystrophic phenotype in *dy^3K^*/*dy^3K^* mice develops around birth and they show a strong hind limb paralysis, but have a shorter life span than *dy^W^*/*dy^W^* mice (16, 19). Because the median survival of the *dy^3K^*/*dy^3K^* mice reaches only 3–5 weeks (16), we compared all genotypes at 4 weeks of age and conducted a second analysis of controls and *dy^3K^*/*dy^3K^* CAG-DL mice at 16 weeks of age. First, we measured laminin-α4 expression in *dy^3K^*/*dy^3K^* mice by western blot analysis of *triceps brachii* muscles from 4-week-old mice. Laminin-α4 was increased to a similar extent as in *dy^W^*/*dy^W^* mice (Fig. S4A), confirming prior data using immunofluorescence on muscle cross-sections (20, 28).

As in *dy^W^*/*dy^W^* mice, expression of the linker proteins strongly improved the overall disease phenotype (Fig. 4E and Movie S2). Until weaning at 3 weeks of age, body mass of *dy^3K^*/*dy^3K^* and *dy^3K^*/*dy^3K^* CAG-DL mice was similar but the two genotypes started to clearly separate at 4 weeks (Fig. 4F). While *dy^3K^*/*dy^3K^* mice did not thrive at all from 2 to 5 weeks of age, controls and *dy^3K^*/*dy^3K^* CAG-DL mice more than doubled their body mass (Fig. 4G, left). The relative increase in body mass from 2 to 12 weeks remained similar between control and *dy^3K^*/*dy^3K^* CAG-DL mice (Fig. 4G, right). These data indicate that the linker proteins do not affect the early disease stage in *dy^3K^*/*dy^3K^* mice until weaning but then allow the mice to thrive both in weight (Fig. 4G) and size (Fig. S4B). Histology of *triceps brachii* muscle was improved in *dy^3K^*/*dy^3K^* CAG-DL mice compared with *dy^3K^*/*dy^3K^* mice (Fig. S4C). The number of muscle fibers as well as their size was increased in *dy^3K^*/*dy^3K^* CAG-DL mice compared with *dy^3K^*/*dy^3K^* mice (Fig. S4D). At 4 weeks of age, mass of TA and *triceps brachii* muscles from *dy^3K^*/*dy^3K^* CAG-DL mice was significantly higher than in *dy^3K^*/*dy^3K^* mice, and muscles of linker-expressing *dy^3K^*/*dy^3K^* mice continued to gain mass (Fig. 4H and I). Similar to in *dy^W^*/*dy^W^* CAG-DL mice, in *dy^3K^*/*dy^3K^* CAG-DL mice, many muscle fibers contained centralized nuclei (Fig. S4E) and showed a strong reduction of fibrosis (Fig. S4F). Functionally, grip strength was significantly higher in 4-week-old *dy^3K^*/*dy^3K^* CAG-DL mice compared with *dy^3K^*/*dy^3K^* mice, and fold increase in grip strength from 4 to 16 weeks was similar between *dy^3K^*/*dy^3K^* CAG-DL and controls (Fig. 4J). When grip strength was normalized to body mass, 4-week-old *dy^3K^*/*dy^3K^* CAG-DL mice were not significantly stronger than *dy^3K^*/*dy^3K^* mice (Fig. S4G), which might be due to the rather late onset of the improvement (see body mass curve in Fig. 4F). However, the normalized forelimb grip strength of *dy^3K^*/*dy^3K^* CAG-DL reached close-to-control levels at the age of 16 weeks (Fig. S4G). Consistent with the improvement in grip strength, ex vivo tetanic and twitch force of EDL and *soleus* muscle was higher in *dy^3K^*/*dy^3K^* CAG-DL mice than in *dy^3K^*/*dy^3K^* mice (Table S1) and increased in *dy^3K^*/*dy^3K^* CAG-DL mice from 4 to 16 weeks. Finally, the overall phenotypic improvement of *dy^3K^*/*dy^3K^* CAG-DL was maintained for a many months without any signs of hind limb paralysis (Movie S3; 2-month-old and 16-month-old *dy^3K^*/*dy^3K^* CAG-DL mice). Taken together, these data demonstrate that the linker proteins largely restore the functionality of skeletal muscle and the peripheral nerve in the two most severe mouse models for LAMA2 MD.

## Discussion

This work shows that ubiquitous expression of the two linker proteins mag and αLNNd is able to correct the disease phenotype in both the skeletal muscle and the peripheral nerve of laminin-α2–deficient mice (*dy^W^*/*dy^W^* and *dy^3K^*/*dy^3K^*). We analyzed the histology of the sciatic nerves only in *dy^W^*/*dy^W^* mice as they allowed us to compare all genotypes. In contrast, dy*^3K^*/*dy^3K^* often die at an age at which axonal sorting is not yet finished (see below), thus preventing us to compare them with *dy^3K^*/*dy^3K^* CAG-DL mice. However, the fact that *dy^3K^*/*dy^3K^* CAG-DL mice did not show any hind limb paralysis (e.g. Movie S3) strongly suggests that the peripheral neuropathy is also restored in this mouse model.

The peripheral neuropathy manifests as progressive hind limb paralysis and hence strongly contributes to the overall phenotype in LAMA2 MD mouse models. Mechanistic studies have shown that this phenotype is likely based on the failure to properly sort the axons according to their size, a process that is driven by Schwann cells and starts in rodents in utero and lasts until a few weeks after birth (25). We examined the peripheral nerves of 8-week-old mice, a time point at which axonal sorting is completed. We find that the linker proteins also prevent reoccurrence of a nerve pathology for a prolonged time as indicated by the rotarod performance of *dy^W^*/*dy^W^* CAG-DL at the age of 5 months and the overall locomotory behavior of a 16-month-old *dy^3K^*/*dy^3K^* CAG-DL mouse. As muscle-specific transgenic expression of the linker proteins already increases median survival of *dy^W^*/*dy^W^* mice from less than 4 months to more than 18 months (15), we did not determine survival of *dy^W^*/*dy^W^* CAG-DL or *dy^3K^*/*dy^3K^* CAG-DL mice. However, our mouse colony includes several *dy^3K^*/*dy^3K^* CAG-DL mice that are older than 1 year, indicating that the linker proteins also have a tremendous beneficial effect on survival in *dy^3K^*/*dy^3K^* mice that have a reported median life span between 3 and 5 weeks (16, 19).

We know from previous studies that expression of both linker proteins adds benefit to skeletal muscle compared with the expression of only one (15). In the current study, we did not test whether this is also the case for the peripheral nerve. Results using adeno-associated virus (AAV)-based delivery of mag in *dy^W^*/*dy^W^* mice and using CMV or hybrid versions of CMV enhancer and chicken beta-actin (CB) promoters to drive expression suggest that mag participates in the improvement of the nerve pathology (29). On the other hand, evidence from LAMA2 MD mouse models that express truncated forms of laminin-α2 suggests a major role of laminin-211 polymerization in axonal sorting. The *dy^2J^*/*dy^2J^* mice express reduced levels of an amino-terminally truncated laminin-α2 subunit (30), and the *dy^nfm417^*/*dy^nfm417^* mice carry a point mutation causing the conversion of an essential cysteine residue to arginine in the laminin N-terminal (LN) domain (required for polymerization) of laminin-α2 (31). Both LAMA2 MD mice present with a severe hind limb paralysis but a much less severe muscular dystrophy than *dy^W^*/*dy^W^* or *dy^3K^*/*dy^3K^* mice. Hence, the peripheral neuropathy in those mouse models is likely based on the compromised polymerization of the mutated laminin-211. Indeed, expression of a shortened version of αLNNd with the ubiquitous hybrid versions of CMV enhancer and chicken beta-actin (CBh) promoter via AAV-mediated delivery does also correct hind limb paralysis in *dy^2J^*/*dy^2J^* mice (32). It remains to be tested whether a single linker protein might be sufficient to prevent nerve pathology in the severe LAMA2 MD mouse models investigated here.

The additive effect of the two linker proteins in skeletal muscle is based on the laminin-polymerizing activity of αLNNd and the α-dystroglycan binding of mag. The two main muscle fiber laminin-211 receptors are α7β1 integrins and dystroglycan (which is posttranslationally cleaved into the ligand-binding α-dystroglycan and the transmembrane β-dystroglycan), both of which cannot bind to laminin-411 (Fig. S5). There is evidence for a functional redundancy of integrin α7β1 and dystroglycan in skeletal muscle (33), and only dystroglycan seems involved in anchoring laminin-211 to the muscle fiber sarcolemma (34). Hence, restoration of the binding of laminin-411 to dystroglycan by mag (which does not bind to α7β1 integrin) may suffice to fully compensate the loss of laminin-211. In the peripheral nerve, loss of laminin-α2 is also compensated by the expression of laminin-α4 (Fig. 1A). In contrast to skeletal muscle, the laminin-β chain that associates with laminin-α4 in the endoneurial BM is not characterized (indicated as X in laminin-4X1 or laminin-2X1; Fig. S5) and thus its binding properties to BM receptors are not well characterized. The nerve pathology in LAMA2 MD mouse models is caused at several steps of Schwann cell–mediated axonal sorting and myelination (25). Deposition of the endoneurial BM is a first step necessary for the entire axonal sorting process. Hence, *Lama2* mutant mice that lack the LN domain of laminin-211, such as *dy^2J^*/*dy^2J^* and *dy^nfm417^*/*dy^nfm417^* mice, likely fail to assemble this BM and hence show a severe nerve pathology, and this function can be restored by polymerizing laminin-411 via αLNNd or a shorter version thereof (32). The Schwann cell receptors of laminin-2X1 involved in axonal sorting are (as for muscle fibers) dystroglycan and integrins. Based on mouse knockout studies, it is proposed that the two receptors are not redundant; integrins are essential in very early steps whereas dystroglycan is required in later stages of axonal sorting (35). Knockout studies in mice showed that the integrin β1 subunit is essential, whereas there is redundancy between several integrin α subunits (13). These experiments show the involvement of different αXβ1 integrins in axonal sorting (Fig. S5). Thus, laminin-4X1 may bind to some of the αXβ1 integrins and hence fully compensate for the loss of laminin-2X1 in the early steps of axonal sorting. In contrast, the binding of mag to α-dystroglycan may allow restoration of the late steps in the axonal sorting process. Based on this hypothesis, both linker proteins would also be required to fully restore the nerve pathology in LAMA2 MD mouse models that are deficient for laminin-α2.

The sequence coding for mag or αLNNd is small enough to be packaged into AAV vectors, which have a maximal capacity for foreign DNA of approximately 5 kb. Thus, AAV-mediated delivery of the two linker proteins might be a possible way to treat LAMA2 MD patients. Our finding that ubiquitous expression of the two linker proteins ameliorates both the muscle and nerve phenotype suggests that such a gene therapeutic approach using a ubiquitous promoter to drive expression of mag and αLNNd might be feasible. Although axonal sorting in the peripheral nerve starts prenatally in mice (25), the work by McKee and Yurchenco (32) shows that intravenous injection of AAV expressing a linker that allows polymerization after birth (postnatal day 1) can still prevent many (if not all) pathological changes in the peripheral nerve of mice with polymerization deficits of laminin-211.

However, null mutations of *LAMA2* in humans manifest largely as a muscular dystrophy and there are only few patients that are reported to suffer from a peripheral neuropathy (36). Thus, muscle-specific promoters to drive expression of the linker proteins may have the advantage of causing less off-target toxicity of the transgenes. It is of course possible that the treatment of the LAMA2 MD patients and the subsequent amelioration of the muscular dystrophy by the linker proteins may make the peripheral neuropathy more apparent. Whether it is an axonal sorting defect that underlies these occasional peripheral neuropathies in human patients remains an open question, largely due to the lack of nerve biopsies from LAMA2 MD patients. Axonal sorting defects have only recently been described in autopsy samples from infantile tissue of spinal muscular atrophy (SMA) patients (37). In SMA, an AAV9-based gene therapy using a ubiquitous promoter to express the missing SMN1 protein was recently approved (38). Although the cells that are involved in the axonal sorting defect differ between SMA (motor neurons) and LAMA2 MD (Schwann cells), the human data from SMA patients suggest that early AAV-based intervention may be important to prevent this sorting defect. Hence, besides the treatment of the muscular dystrophy, early treatment of LAMA2 MD patients may also prevent a possible axonal sorting defect.

Recent evidence from gene therapy trials in Duchenne muscular dystrophy (DMD) patients indicate that the expression of microdystrophin may cause some immune reaction against the transgene (39). In contrast to the situation in DMD where patients do not express dystrophin, mag and αLNNd are assembled from domains of proteins that are also expressed by LAMA2 MD patients and thus they should not cause strong immune responses. Moreover, both linker proteins are secreted from the cells that express them and exert their function in the extracellular matrix. Hence, they can also be incorporated into the BM adjacent to nonexpressing cells. This feature may also be a substantial advantage in gene therapeutic approaches as it may allow lowering the dose needed to treat patients. In summary, the data presented here using the newly generated transgenic mice further support the concept of AAV-based gene therapy as a viable treatment option for LAMA2 MD patients. Our data open the possibility to use ubiquitous expression of the linker proteins so that all tissues that might be affected in the disease are reached.

## Materials and methods

### Mice

As mouse models for LAMA2 MD, we used *dy^W^*/*dy^W^* mice (22, 40) [B6.129S1(Cg)-*Lama2^tm1Eeng^*/J; available from the Jackson Laboratory stock #013786] and *dy^3K^/dy^3K^* mice [B6.129P2(Cg)-*Lama2^tm1Stk^*, a kind gift from Drs. Shin'ichi Takeda and Yuko Miyagoe-Suzuki] (27). Genotyping was performed as described previously for *dy^W^*/*dy^W^* mice (22) and *dy^3K^/dy^3K^* mice (20).

Transgenic knockin mag mice [C57BL/6J-Gt(ROSA)26Sor^tm1(CAG-mag)Rueg^] and αLNNd mice [C57BL/6J-Gt(ROSA)26Sor^tm2(CAG-aLNNd)Rueg^] were generated by CRISPR-Cas9–mediated knockin into the *Rosa26* locus. To make mag resistant to protein cleavage, we introduced the single amino acid substitution K793A into the sequence encoding the mouse version of mag (26) by PCR-based site-directed mutagenesis. Nucleotide sequences for mag and αLNNd (21) were subcloned by Gibson Assembly into the Ai9 Rosa26 targeting vector (23) (Addgene Cat. 22799) by replacing tdTomato. The diphtheria toxin cassette in the Ai9 plasmid was removed by restriction digest.

Transgene integration was carried out using targeted integration with linearized dsDNA CRISPR [Tild-CRISPR (41)] in fertilized mouse oocytes. Linearized dsDNA fragments used for targeting contained the transgene cassette flanked by ∼800-bp-long homology arms targeting the canonical *Xba*I site in the first intron of the *Rosa26* locus. crRNA targeting the overlapping sequence 5′-ACT CCA GTC TTT CTA GAA GA**T GG** [*Xba*I site underlined and PAM sequence in bold; sequence according to sgRosa26–1 described previously (42)] was used to generate cr:trcrRNA-Cas9 ribonucleoproteins (RNPs) capable of inducing DNA double-strand breaks and subsequent homologous recombination at the target site. C57BL/6J female mice underwent ovulation induction by intraperitoneal (i.p.) injection of 5 IU equine chorionic gonadotrophin (PMSG; Folligon–InterVet), followed by i.p. injection of 5 IU human chorionic gonadotrophin (Pregnyl–Essex Chemie) 48 h later. For the recovery of zygotes, C57BL/6J females were mated with males of the same strain immediately after the administration of human chorionic gonadotrophin. All zygotes were collected from oviducts 24 h after the human chorionic gonadotrophin injection and were then freed from any remaining cumulus cells by a 1–2-min treatment of 0.1% hyaluronidase (Sigma-Aldrich) dissolved in M2 medium (Sigma-Aldrich). Mouse embryos were cultured in M16 (Sigma-Aldrich) medium at 37°C and 5% CO_2_. For micromanipulation, embryos were transferred into M2 medium. All microinjections were performed using a microinjection system comprised of an inverted microscope equipped with Nomarski optics (Nikon), a set of micromanipulators (Narashige), and a FemtoJet microinjection unit (Eppendorf). Injection solution containing 100 ng/*µ*L (60 *µ*m) Cas9 protein (IDT), 100 *µ*m cr:trcrRNA Rosa26 (IDT), and 20 ng/*µ*L linearized dsDNA was microinjected into the male pronuclei of fertilized mouse oocytes until 20–30% distension of the organelle was observed. Embryos that survived the microinjection were transferred on the same day into the oviducts of 8–16-week-old pseudopregnant Crl:CD1 (ICR) females (0.5 day used after coitus) that had been mated with sterile, genetically vasectomized males (43) the day before embryo transfer. Pregnant females were allowed to deliver and raise their pups until weaning age.

Targeted pups were screened by PCR with primers amplifying the transgenes mag or αLNNd. Correct targeting was further confirmed with a PCR using a primer recognizing a genomic sequence outside of the upstream homology region of the targeting vector and a second primer in mag or αLNNd. Targeted founder mice were crossed to homozygosity, and loss of wild-type *Rosa26* allele was confirmed by PCR. Genotyping was performed with primers on the *Rosa26* wild-type allele: 5′-AAG GGA GCT GCA GTG GAG TA and 5′-CCG AAA ATC TGT GGG AAG TC; on αLNNd: 5′-AGC TGA TCC GGA ACC CTT AA and 5′-GGA TGG CGC TCT CTA GGA TT; and on mag: 5′-AAG GGA GCT GCA GTG GAG TA and 5′-CCG AAA ATC TGT GGG AAG TC.

To express mag and αLNNd ubiquitously, mice were crossed with CAG-Cre-ER mice (24) [B6.Cg-Tg(CAG-cre/Esr1*)5Amc/J available from the Jackson Laboratory stock 004682]. CAG-Cre-ER mice were crossed with tdTomato-expressing reporter mice [Ai9; B6;129S6-Gt(ROSA)26Sortm9(CAG-tdTomato)Hze/J (23) available from the Jackson Laboratory] to control for Cre-mediated recombination in skeletal muscle. For tamoxifen application, tamoxifen (Sigma; T5648-1G) was dissolved in corn oil (Sigma; C8267) at a concentration of 20 mg/mL by shaking overnight at 37°C and administrated at a dose of 75 mg/kg by i.p. injection once every 24 h for a total of 5 consecutive days. Fourteen days after the first injection, tissue was collected and recombination and removal of the LSL cassette were confirmed on purified DNA using primers flanking the LSL cassette: 5′-GCT GGT TAT TGT GCT GTC TCA TC and 5′-TGC ACT TAA CGC GTA CAA GG.

All mice analyzed were from breedings of mice heterozygous for the knockout allele in the *Lama2* locus, hemizygous for CAG-Cre-ER, and homozygous for LSL-αLNNd or LSL-mag alleles, respectively. This strategy allowed to receive all genotypes from the same breeding and use littermates as controls. Control mice were always Cre-negative and heterozygous for LSL-αLNNd and LSL-mag; for the *Lama2* locus, controls were either wild-type or heterozygous. Unless otherwise indicated, female and male mice were used. To ensure optimal access of the dystrophic mice to water and food, all cages were supplied with long-necked water bottles and wet food from weaning onwards. All mouse experiments were performed according to the federal guidelines for animal experimentation and approved by the authorities of the Canton of Basel-Stadt.

### Antibodies

For immunostaining and western blot analysis, the following antibodies were used: α-actinin (Sigma, catalog no. A7732; 1:5,000), agrin [for detection of mag; produced in-house (44); 1:5,000 for western blots and 1:200 for immunostainings or R&D System catalog no. AF550; 5 *µ*g/mL for immunostainings], laminin-α1 (for detection of αLNNd by western blot; R&D Systems, catalog no. AF4187; 1:2,000), αLNNd [for detection of αLNNd by immunostainings, previously described (21); 1:100], laminin-α2 (Sigma, catalog no. L0663, clone 4H8-2; 1:500), laminin-α4 [previously described (21); 1:1,000 for western blots and 1:200 for immunostainings], laminin-β1γ1 (Sigma, catalog no. L9393; 1:100), and GAPDH (Cell Signaling, catalog no. 2118, 1:1,000).

### Immunostainings

Fresh frozen muscle or sciatic nerve tissue was cryosectioned (10 *μ*m) and fixed with 4% paraformaldehyde (PFA) in phosphate-buffered saline (PBS) (4% PFA/PBS) or stained nonfixed. Sections were incubated in blocking solutions (5% donkey serum and 0.3% Triton-X in PBS) for 1 h at room temperature and then incubated overnight at 4°C with primary antibodies, diluted in 5% donkey serum in PBS. Sections were washed three times with PBS and incubated at room temperature for 1 h with the appropriate secondary antibodies. Images were acquired with an Olympus iX81 microscope, a Zeiss Axio Scan.Z1 Slide Scanner, or a Zeiss point scanning Confocal microscope LSM700.

### Protein extraction and western blot analysis

For total muscle extracts, frozen muscles were pulverized in liquid nitrogen, lysed in modified radioimmunoprecipitation assay (RIPA) buffer (50 mm Tris-HCl, pH 8.0, 150 mm NaCl, 1% NP-40, 0.5% deoxycholate, 0.1% SDS, and 20 mm EDTA; protease inhibitors), sonicated, and incubated for 2 h at 4°C with head-over-head rotation. Insoluble material was removed by centrifugation (16,000 g, 30 min, 4°C). After adjustment of protein concentrations (determined by bicinchoninic acid (BCA) assay), Laemmli buffer was added and samples were incubated for 5 min at 95°C and subjected to standard western blot procedures.

### Histology and histological quantifications of muscle

Muscles were mounted on 7% gum tragacanth (Sigma) and rapidly frozen in 2-methylbutane that was cooled in liquid nitrogen (−150°C). Cross-sections of 10 *μ*m thickness were cut on a cryostat. General histology was assessed after tissue fixation with 4% paraformaldehyde by H&E staining (Merck) or Picro Sirius Red stain [Direct Red 80 (Sigma) in picric acid solution]. Images were acquired with an Olympus iX81 microscope using cellSens software (Olympus).

The muscle fiber diameter was quantified using the minimum distance of parallel tangents at opposing particle borders (minimal “Feret's diameter”), as described previously (45). For fiber number, fiber size, and CNF analysis, complete midbelly cross-sections were evaluated using an in-house–customized, Fiji-based version of Myosoft (46, 47). The algorithms used are available at 10.5281/zenodo.6469872.

### Hydroxyproline quantification

Fibrosis in muscles was determined by measuring the amount of the collagen-specific amino acid hydroxyproline. Frozen muscles were pulverized in liquid nitrogen and dried with SpeedVac. Mass spectrometry–based amino acid analysis was performed at Functional Genomics Center Zürich, ETH Zürich/University of Zürich, Zürich, Switzerland. In brief, samples were dissolved in 0.1 N acetic acid, mixed for 3 h at room temperature, and dried by SpeedVac. Samples were hydrolyzed with 6 m HCl under 0.1% w/v phenol stream at 110°C for 24 h and then dried using SpeedVac. Labeling was performed with aminoquinolyl-*N*-hydroxysuccinimidyl carbamate (Waters) and samples measured against a standard curve containing Amino Acid Standard H (Waters) and 4-hydroxyproline (Agilent) and with an internal standard ^13^C–^15^N-labeled MSK-A (Cambridge Isotopes Laboratories).

### Histology and morphometric analysis of sciatic nerves

Semithin and ultrathin morphological experiments were performed as described previously (48). In brief, sciatic nerves were dissected and fixed with 2.5% glutaraldehyde in 0.12 m phosphate buffer pH 7.4, postfixed with 1% osmium tetroxide, and embedded in epon (Sigma catalog no. 45359). Semithin sections (1 *µ*m) were stained with 0.1% toluidine blue and examined by light microscopy on a BX51 Olympus microscope. To perform morphometric analysis, images of cross-sections were obtained from corresponding levels of the sciatic nerve by a digital camera (DCF7000T, Leica) with 100× objective. Five images per animal were acquired and analyzed using the Leica QWin Software (Leica Microsystem, Milan). The *G*-ratio (axon diameter/fiber diameter) was determined by dividing the mean diameter of an axon by the mean diameter of the same fiber (axon plus myelin). There were 350–600 myelinated fibers per mouse quantified corresponding to all the myelinated fibers present in five random images (at 100× images). Ultrathin sections (70–80 nm thick) were stained with uranyl acetate and lead citrate and examined by electron microscopy (FEI Talos L1200C G2 Transmission Electron Microscope). At least 20 images per animal, randomly photographed, were acquired and analyzed using ImageJ (National Institutes of Health) software.

### Cell culture

COS7 cells were cultured in Dulbecco's Modified Eagle's Medium (DMEM) medium (Invitrogen) in the presence of 10% fetal bovine serum (Gibco). Transfections of LSL-mag, LSL-αLNNd, and CAG-Cre (49) (Addgene Cat. 13775) plasmids were performed with Lipofectamine 2000 (Invitrogen) following the manufacturer's instructions. For the analysis of proteins in cell-conditioned medium, medium was replaced 24 h after transfection with serum-free DMEM. Forty-eight hours after transfection, conditioned medium was collected and COS7 cells were lysed. Both samples were subjected to western blot analysis.

### Grip strength measurement

Forelimb grip strength was assessed according SOP MDC1A_M.2.2.001 (TREAT-NMD) using a grip strength meter (Columbus) equipped with a trapeze bar. Mice were lifted on the tail towards the bar until the mouse gripped it with both forepaws and then gently moved away at constant speed until its grip was lost. The peak force was calculated as the mean of the three best trials out of six consecutive pulls.

### Gait analysis

Gait analysis was performed using the CatWalk XT system (Noldus) following the manufacturer's instructions. Mice were placed in an enclosed illuminated walkway on a glass plate, allowed to move freely in both directions, and their footprints recorded by a high-speed video camera positioned underneath the walkway. Runs were classified as compliant when mice crossed a defined 40 cm distance in the walkway within 10 s and a maximum speed variation of 60%. Three compliant runs were recorded for each mouse and averaged for statistical analysis. For *dy^W^*/*dy^W^* mice, only three out of six mice measured were able to perform compliant runs, whereas compliant runs were obtained for all assessed control and *dy^W^*/*dy^W^* CAG-DL mice.

### Assessment of motor coordination (rotarod assay)

Motor coordination of mice was assessed using a rotarod device (Ugo Basile). Mice were trained for 2 consecutive days by placing them three times for 1 min on the rotarod rotating at a constant speed of 5 rpm. On the third consecutive day, performance was assessed on accelerating rod (5–40 rpm within 5 min) for a maximal duration of 400 s. The test was performed three times with minimal rest period of 10 min between trials. The time on the rod (latency to fall) was averaged for the three trials.

### In vitro muscle force measurement

In vitro muscle force measurements were performed on isolated EDL or *soleus* muscle using the 1200A Isolated Muscle System (Aurora Scientific) in an organ bath at 30°C containing Ringer solution (137 mm NaCl, 24 mm NaHCO_3_, 11 mm glucose, 5 mm KCl, 2 mm CaCl_2_, 1 mm MgSO_4_, and 1 mm NaH_2_PO_4_) constantly oxygenated with 95% O_2_/5% CO_2_. After muscles were adjusted to the optimum muscle length (Lo), muscles were stimulated with electrical pulse at 15 V and achieved peak twitch force (Pt) recorded. Peak tetanic force (Po) was assessed as maximal force with 500 ms stimulation at 10–250 Hz. Specific twitch and tetanic forces were calculated by normalization to the cross-sectional area (CSA) by using the formula CSA (mm^2^) = muscle wet weight (mg)/[fiber length (lf, mm) × 1.06 mg/mm^3^], with lf = lo × 0.44 for EDL or lf = lo × 0.71 for *soleus* as described (50).

### Study design and statistical analysis

Mice were randomly allocated to experimental groups. Evaluations of immunohistochemistry, muscle histology, muscle function, and behavior assays were performed by investigators blinded to the specific sample. Statistical analysis was performed using unpaired, two-tailed Student's *t* test for comparisons of two groups. For the comparisons of more than two groups, one-way ANOVA followed by Bonferroni post hoc test was used or, if unequal variance between groups was detected, one-way ANOVA with Dunnett's multiple comparisons test. We assumed normal distribution of the variables analyzed. All statistical tests were performed using Prism version 9 (GraphPad Software).

## Supplementary Material

pgad083_Supplementary_Data

## Data Availability

All data are included in the article and Supplementary Material.

## Preprints

A previous version of this manuscript was posted on a preprint server: https://doi.org/10.1101/2022.05.19.492629.
