# Supplementary material for: Nerve pathology is prevented by linker proteins in mouse models for LAMA2-related muscular dystrophy
Source: PNAS Nexus. 2023 Mar 15;2(4):pgad083. doi: 10.1093/pnasnexus/pgad083 (PMC10082391; doi:10.1093/pnasnexus/pgad083)
Supplement: pgad083_Supplementary_Data [file pgad083_supplementary_data.zip › PNASNEXUS-PNASNEXUS-2022-00795-T-s01.docx]

**
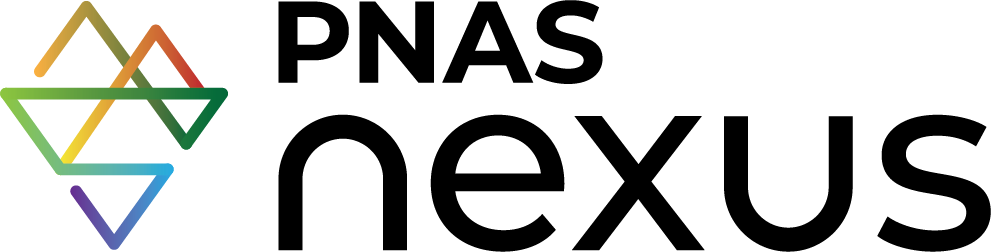
**

**Supplementary Information for**

Nerve pathology is prevented by linker proteins in mouse models for *LAMA2*-related muscular dystrophy

Judith R. Reinhard, Emanuela Porrello, Shuo Lin, Pawel Pelczar, Stefano C. Previtali and Markus A. Rüegg

*Corresponding authors email: [markus-a.ruegg@unibas.ch](mailto:markus-a.ruegg@unibas.ch) and [judith.reinhard@unibas.ch](mailto:judith.reinhard@unibas.ch)

**This PDF file includes:**

Figures S1 to S5

Table S1

Legends for Movies S1 to S3

**Other supplementary materials for this manuscript include the following:**

Movies S1 to S3


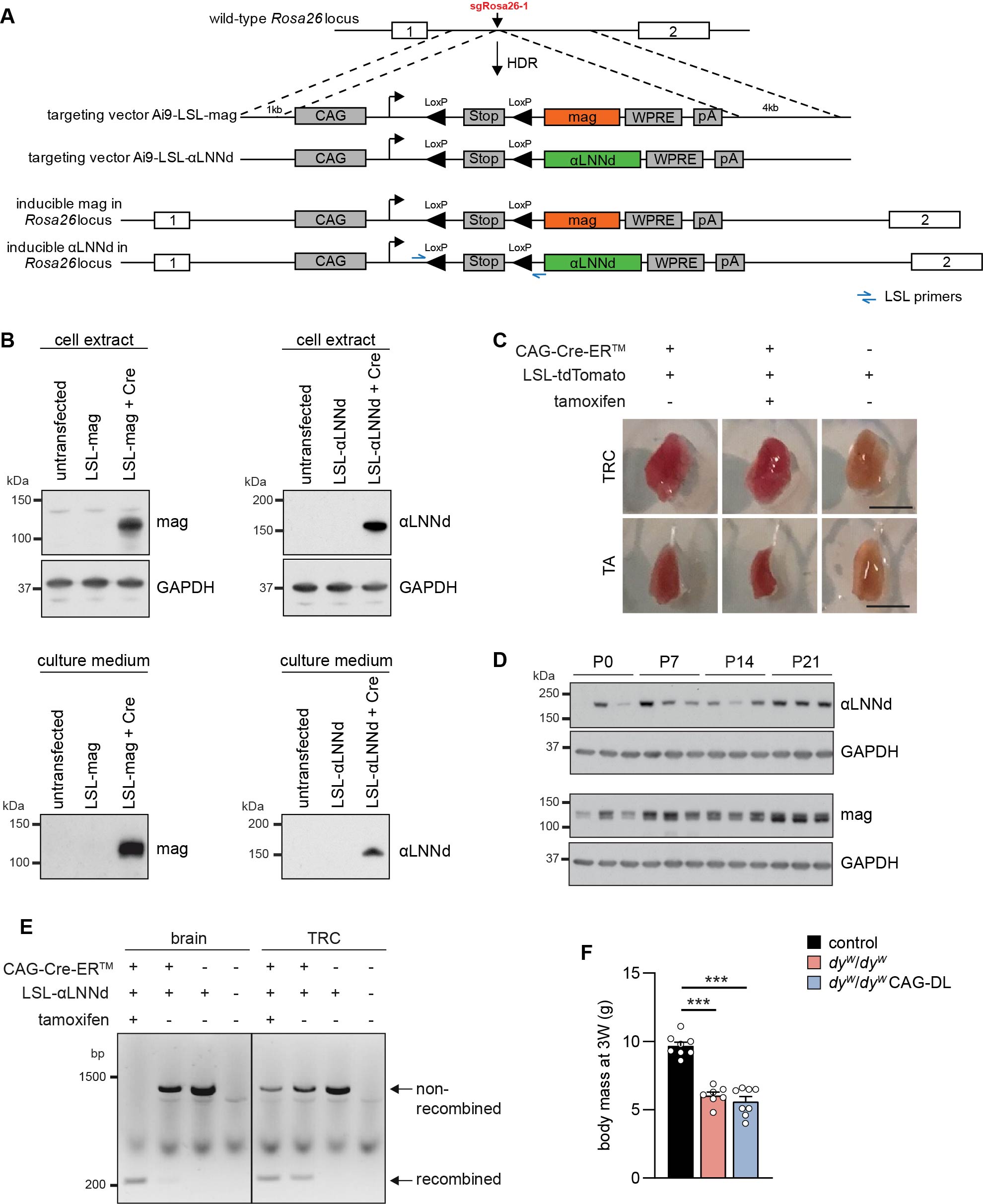


**Fig. S1. Generation and characterization of mice expressing the linker proteins ubiquitously (CAG-DL mice).**

**A** Schematic of the Cre-dependent LSL-mag and LSL-αLNNd targeting constructs and the position of the sgRosa26-1 used for cutting the *Rosa26* locus.

**B** Western blot analysis of cell extracts and cell culture medium from COS7 cells transfected with indicated plasmids. GAPDH was used as loading control.

**C** Expression of tdTomato in *triceps brachii* (TRC) and *tibialis anterior* (TA) muscle of 5-week-old mice with the indicated genotype and upon injection of tamoxifen. Note that tdTomato is expressed in both muscles irrespective of tamoxifen treatment.

**D** Western blot analysis of lysate from TRC muscle of CAG-DL mice not injected with tamoxifen from postnatal day zero (P0) to P21. GAPDH was used as loading control.

**E** PCR on genomic DNA purified from brain or TRC muscle from 8-week-old mice using the indicated primers in (**A**). PCR products before (non-recombined) and after Cre-mediated recombination (recombined) of the LSL cassette are indicated. Note the high level of recombination observed in TRC muscle in CAG-CreER^TM^-positive mice that were not treated with tamoxifen.

**F** Body mass of 3-week-old mice of the indicated genotypes.

Data are mean ± SEM. ****P* < 0.001; by one-way ANOVA with Bonferroni post hoc test. Scale bars: 5 mm. N = 7-8 mice per group. Type or paste legend here. Paste figure above the legend.

**
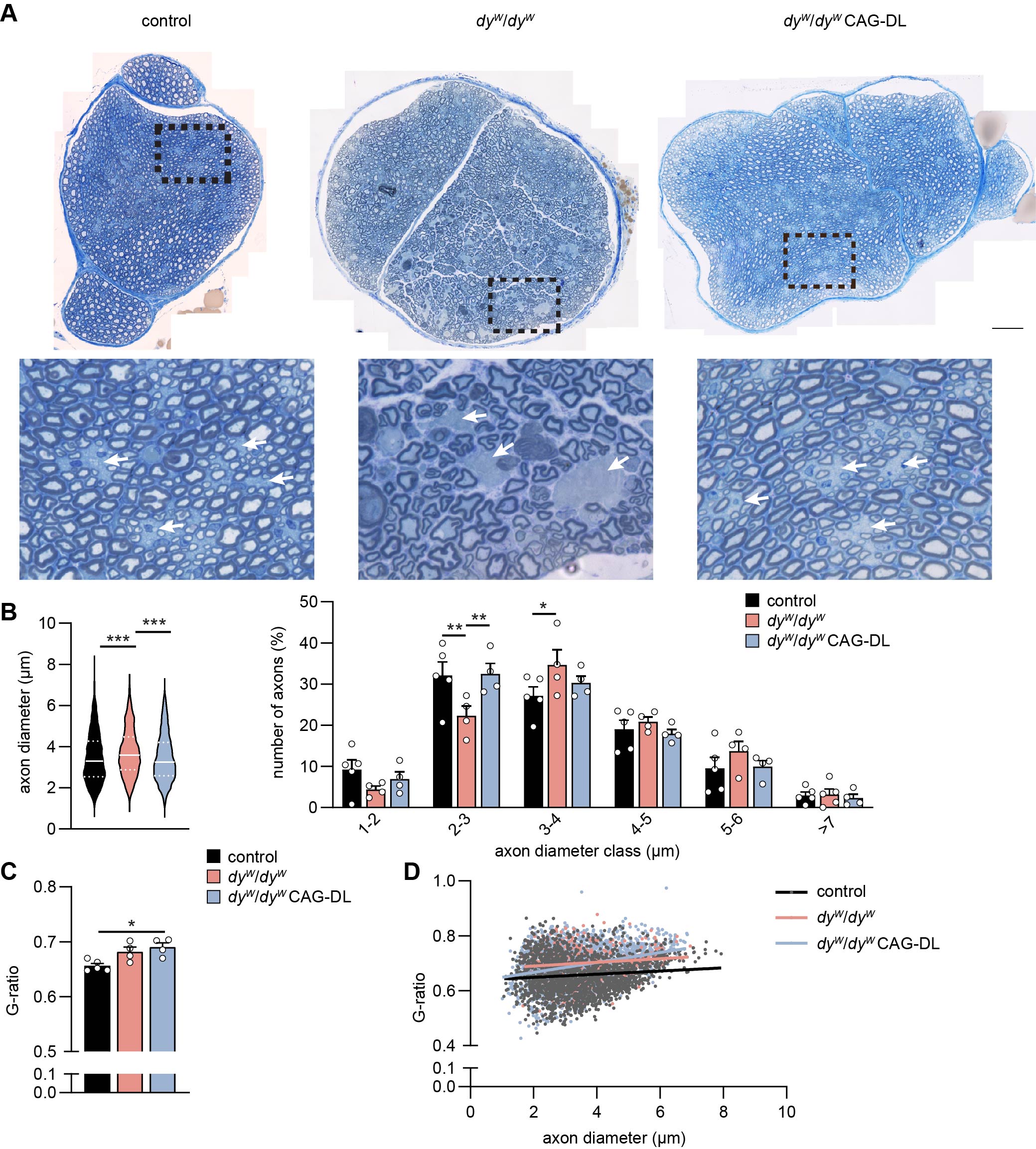
Fig. S2. Quantification of nerve pathology.**

**A** Reconstruction of the entire nerve using toluidine blue-stained, semi-thin sections of the sciatic nerve from 8-week-old mice of the indicated genotype. Arrows point towards bundles of non-myelinated axons. The area occupied by these bundles is increased in *dy^W^*/*dy^W^* mice compared to control and *dy^W^*/*dy^W^* CAG-DL mice. Regions with non-myelinated axons in *dy^W^*/*dy^W^* CAG-DL mice are reminiscent of Remak bundles observed in controls.

**B** Quantification of axon diameter of myelinated axons of the sciatic nerve from 8-week-old mice of the indicated genotype shown as violin blot of all measured fibers (left) and size distribution per mouse (right).

**C-D** Quantification of myelination in sciatic nerves from 8-week-old mice of the indicated genotype by the mean G-ratio (**C**) and the scatter plot of G-ratios versus axon diameter (**D**).

Data are mean ± SEM. **P* < 0.05; ***P* < 0.01; ****P* < 0.001; by one-way ANOVA with Bonferroni post hoc test. For violin blot in **B** horizontal lines indicate median and 25^th^ and 75^th^ percentile. Lines indicate the linear regression in **D**. N = 4-5 mice per group (350-600 fibers per mouse). Scale bar: 40 µm.


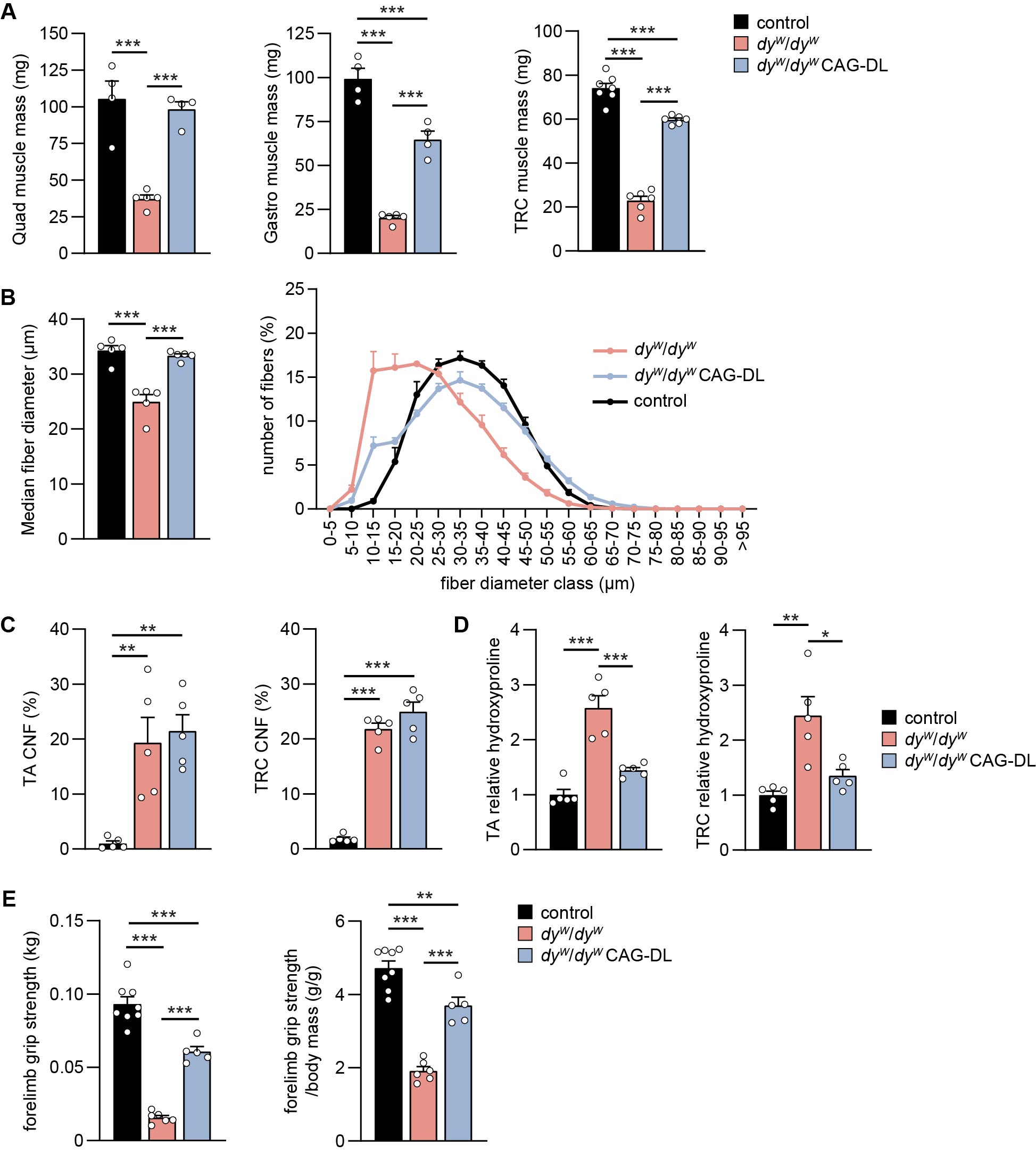


**Fig. S3. Quantification of muscle histology grip strength.**

**A** Quantification of muscle mass of *quadriceps* (Quad), *gastrocnemius* (Gastro) and *triceps brachii* (TRC) of 8-week-old female mice of the indicated genotype.

**B** Quantification of muscle fiber diameters of the TRC from 8-week-old female mice of the indicated genotype.

**C** Quantification of fibers with centralized nuclei (CNF) of TA and TRC muscle.

**D** Quantification of fibrosis by amino acid analysis of the hydroxyproline content of TA and TRC muscle.

**E** Grip strength performance of 8-week-old female mice of the indicated genotype.

Data are mean ± SEM. **P* < 0.05; **P* < 0.005; ****P* < 0.001; by one-way ANOVA with Bonferroni post hoc test. N = 4-8 mice per group.


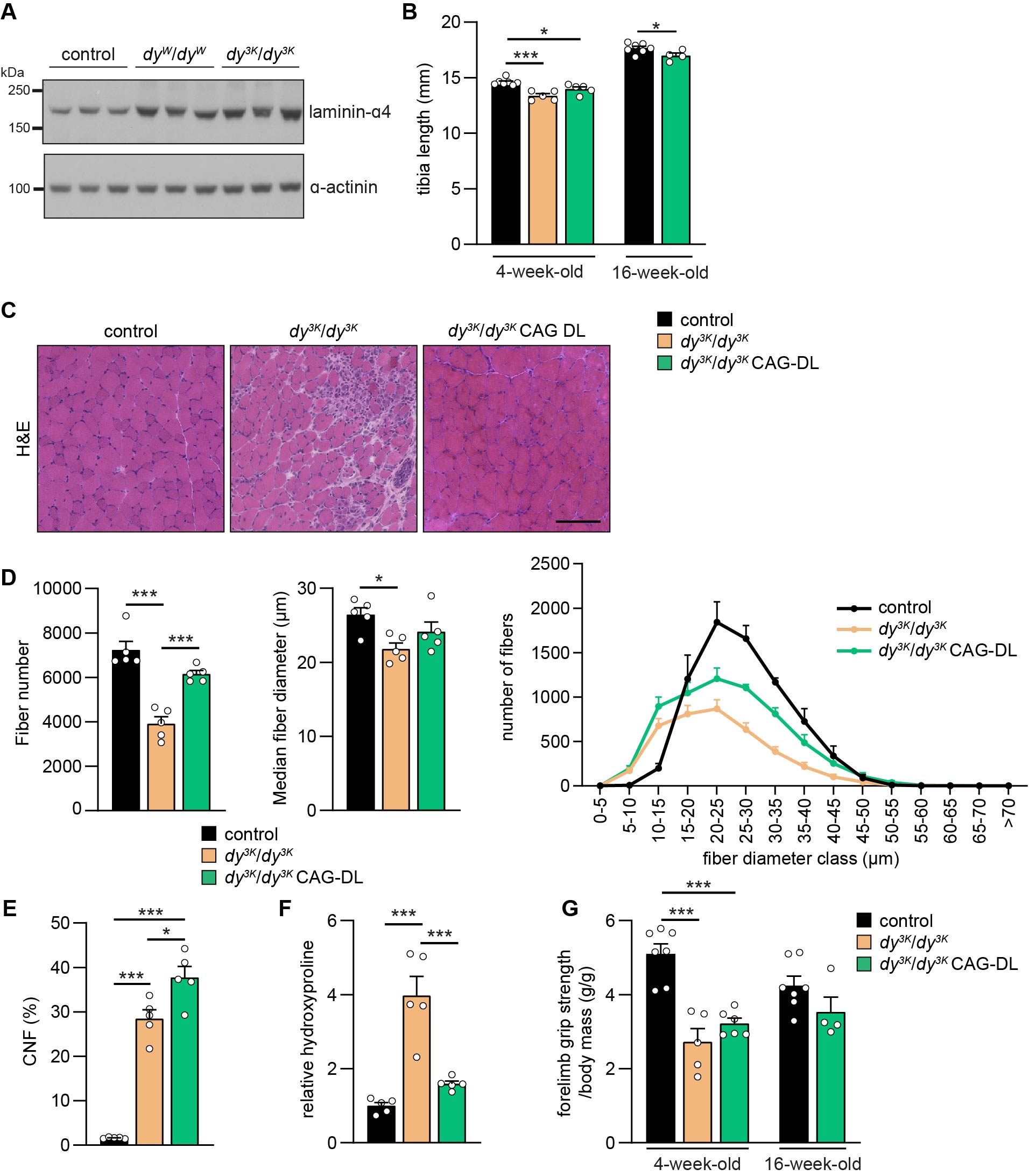


**Fig. S4. Treatment effect in *dy^3K^*/*dy^3K^* mice.**

**A** Western blot analysis for laminin-α4 in 4-week-old mice of the indicated genotype. α-actinin was used as loading control.

**B** Tibia length of 4- and 16-week-old mice of the indicated genotype.

**C** Hematoxylin and Eosin (H&E) of 4-week-old *triceps brachii* muscle of the indicated genotype.

**D** Quantification of *triceps brachii* muscle fiber number and diameter of 4-week-old female mice.

**E** Quantification of fibers with centralized nuclei (CNF) of *triceps brachii* muscle of 4-week-old mice.

**F** Quantification of fibrosis by amino acid analysis of hydroxyproline content of *tibialis anterior* muscle from 4-week-old mice.

**G** Grip strength performance normalized to body mass of 4- and 16-week-old female mice of the indicated genotype.

Data are mean ± SEM. **P* < 0.05; **P* < 0.005; ****P* < 0.001; by one-way ANOVA with Bonferroni post hoc test. Scale bar: 100 µm. N = 4-7 mice per group.


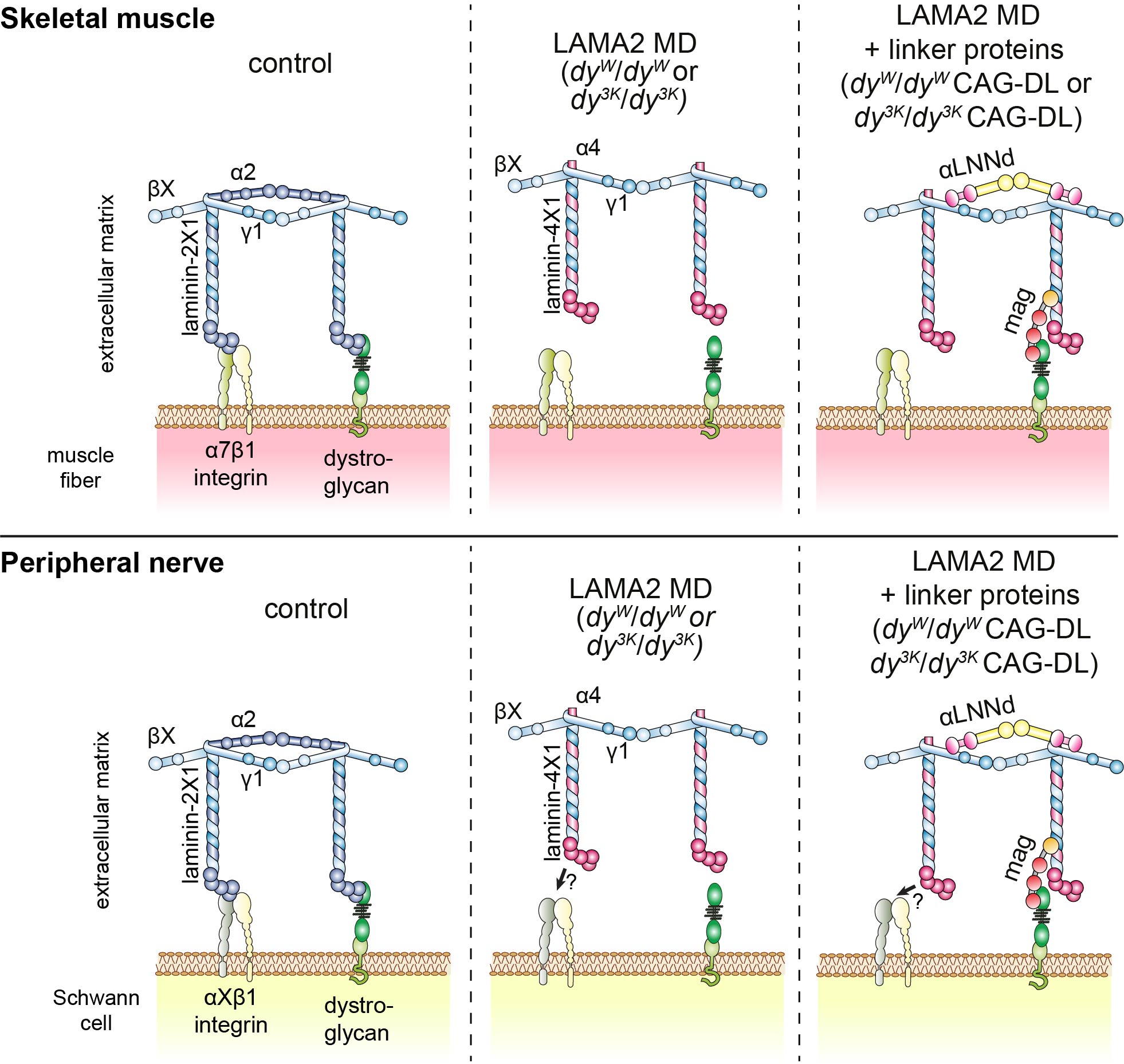


**Fig. S5. Figure S5. Model depicting the function of linker proteins in skeletal muscle and peripheral nerve.**

(Top) In normal skeletal muscle laminin-2X1 (two possible β-chains) is anchored to the muscle fibers membrane via α7β1-integrin and α-dystroglycan. Lack of laminin-2X1 causes compensatory upregulation on laminin-4X1, which does not bind to α7β1-integrin and α-dystroglycan and is not polymerizing. Presence of αLNNd allows laminin-411 to polymerize and mag bridges to α-dystroglycan.

(Bottom) In normal peripheral nerve laminin-2X1 (two possible β-chains) is linked to α-dystroglycan and αXβ1-integrin (various possible α-subunits). Laminin-4X1 (two possible β-chains) fails to bind α-dystroglycan and eventually integrin (depending on the integrin α-subunit present). Similarly as in skeletal muscle, linker proteins prevent nerve pathology by allowing polymerization (via αLNNd) and linking to α-dystroglycan (via mag).

|  | control  8W  (N=7) | *dy^W^*/*dy^W^*  8W  (N=5) | *dy^W^*/*dy^W^* CAG-DL  8W  (N=5) |
| --- | --- | --- | --- |
| EDL |  |  |  |
| muscle mass (mg) | 6.91 ± 0.26 | 2.84 ± 0.26 | 5.74 ± 0.29 |
| optimal length (mm) | 12.41 ± 0.19 | 9.89 ± 0.45 | 11.43 ± 0.34 |
| Pt (mN) | 38.28 ± 2.13 | 7.96 ± 1.12 | 31.20 ± 2.58 |
| sPt (mN/mm^2^) | 32.12 ± 1.75 | 12.81 ± 1.12 | 29.09 ± 2.35 |
| Po (mN) | 274.4 ± 18.00 | 72.39 ± 5.88 | 212.0 ± 13.22 |
| sPo (mN/mm^2^) | 229.4 ± 12.46 | 118.2 ± 6.20 | 197.1 ± 10.02 |
| *soleus* |  |  |  |
| muscle mass (mg) | 6.11 ± 0.35 | 2.65 ± 0.27 | 4.16 ± 0.20 |
| optimal length (mm) | 10.95 ± 0.21 | 9.01 ± 0.53 | 10.17 ± 0.27 |
| Pt (mN) | 29.91 ± 1.21 | 8.46 ± 0.89 | 14.22 ± 0.83 |
| sPt (mN/mm^2^) | 40.90 ± 2.15 | 21.80 ± 1.36 | 26.54 ± 1.85 |
| Po (mN) | 198.8 ± 11.66 | 62.48 ± 6.82 | 95.97 ± 2.85 |
| sPo (mN/mm^2^) | 269.6 ± 10.41 | 160.2 ± 8.13 | 178.8 ± 5.89 |

|  | control  4W  (N=6) | *dy^3K^*/*dy^3K^*  4W  (N=5) | *dy^3K^*/*dy^3K^* CAG-DL 4W  (N=6) | control  16W  (N=6) | *dy^3K^*/*dy^3K^* CAG-DL 16W  (N=4) |
| --- | --- | --- | --- | --- | --- |
| EDL |  |  |  |  |  |
| muscle mass (mg) | 4.48 ± 0.19 | 1.89 ± 0.33 | 3.61 ± 0.22 | 8.24 ± 0.31 | 7.18 ± 0.57 |
| optimal length (mm) | 10.76 ± 0.24 | 8.34 ± 0.41 | 9.93 ± 0.39 | 13.10 ± 0.08 | 12.52 ± 0.30 |
| Pt (mN) | 28.91 ± 1.59 | 5.28 ± 0.78 | 13.44 ± 2.52 | 45.12 ± 1.03 | 28.50 ± 3.61 |
| sPt (mN/mm^2^) | 32.47 ± 1.69 | 11.17 ± 1.32 | 16.91 ± 3.04 | 33.53 ± 0.58 | 23.06 ± 1.56 |
| Po (mN) | 189.0 ± 7.03 | 38.87 ± 7.88 | 94.76 ± 15.04 | 314.9 ± 9.54 | 198.6 ± 21.74 |
| sPo (mN/mm^2^) | 211.8 ± 4.87 | 78.59 ± 10.92 | 119.2 ± 17.26 | 233.9 ± 5.18 | 161.0 ± 7.95 |
| *soleus* |  |  |  |  |  |
| muscle mass (mg) | 3.78 ± 0.22 | 1.71 ± 0.27 | 2.51 ± 0.19 | 7.56 ± 0.62 | 5.38 ± 0.80 |
| optimal length (mm) | 9.74 ± 0.31 | 7.27 ± 0.31 | 9.14 ± 0.30 | 11.81 ± 0.35 | 11.36 ± 0.22 |
| Pt (mN) | 21.57 ± 1.31 | 5.49 ± 1.22 | 8.12 ± 1.39 | 35.30 ± 0.76 | 15.14 ± 1.565 |
| sPt (mN/mm^2^) | 41.70 ± 1.46 | 16.92 ± 2.06 | 21.98 ± 2.96 | 42.31 ± 2.85 | 24.55 ± 0.92 |
| Po (mN) | 116.5 ± 7.33 | 31.29 ± 7.65 | 45.48 ± 7.57 | 250.9 ± 14.63 | 120.4 ± 15.79 |
| sPo (mN/mm^2^) | 225.3 ± 8.89 | 94.92 ± 14.20 | 123.3 ± 16.46 | 297.9 ± 14.85 | 192.7 ± 1.59 |

**Table S1. Treatment effect on muscle force.**

Quantification of muscle mass, length and force of EDL or soleus muscle of 8-week old female mice (top) or 4-week and 16-week old female mice (bottom) of indicated genotype. Abbreviations used are, Pt: peak twitch force; sPt: specific peak twitch force; Po: peak tetanic force; sPo: specific peak tetanic force. Data are mean ± SEM. N = 4-7 mice per group.

**Movie S1.** 14-week-old *dy^W^*/*dy^W^*, *dy^W^*/*dy^W^* CAG-DL and control mouse.

**Movie S2.** 4-week-old *dy^3K^*/*dy^3K^*, *dy^3K^*/*dy^3K^* CAG-DL and control mouse.

**Movie S3.** 2-month-old and 16-month-old *dy^3K^*/*dy^3K^* CAG-DL mouse.
